# Comparative Brain Stem Lesions on MRI of Acute Disseminated Encephalomyelitis, Neuromyelitis Optica, and Multiple Sclerosis

**DOI:** 10.1371/journal.pone.0022766

**Published:** 2011-08-10

**Authors:** Zhengqi Lu, Bingjun Zhang, Wei Qiu, Zhuang Kang, Liping Shen, Youming Long, Junqi Huang, Xueqiang Hu

**Affiliations:** 1 Department of Neurology, The Third Affiliated Hospital of Sun Yat-sen University, Guangzhou, China; 2 Department of Radiology, The Third Affiliated Hospital of Sun Yat-sen University, Guangzhou, China; 3 Key Laboratory of Tropical Diseases Control, Ministry of Education and Department of Immunology/Institute of Immunology, Zhongshan School of Medicine, Sun Yat-sen University, Guangzhou, China; Institute Biomedical Research August Pi Sunyer (IDIBAPS) - Hospital Clinic of Barcelona, Spain

## Abstract

**Background:**

Brain stem lesions are common in patients with acute disseminated encephalomyelitis (ADEM), neuromyelitis optica (NMO), and multiple sclerosis (MS).

**Objectives:**

To investigate comparative brain stem lesions on magnetic resonance imaging (MRI) among adult patients with ADEM, NMO, and MS.

**Methods:**

Sixty-five adult patients with ADEM (n = 17), NMO (n = 23), and MS (n = 25) who had brain stem lesions on MRI were enrolled. Morphological features of brain stem lesions among these diseases were assessed.

**Results:**

Patients with ADEM had a higher frequency of midbrain lesions than did patients with NMO (94.1% vs. 17.4%, *P*<0.001) and MS (94.1% vs. 40.0%, *P*<0.001); patients with NMO had a lower frequency of pons lesions than did patients with MS (34.8% vs. 84.0%, *P*<0.001) and ADEM (34.8% vs. 70.6%, *P* = 0.025); and patients with NMO had a higher frequency of medulla oblongata lesions than did patients with ADEM (91.3% vs. 35.3%, *P*<0.001) and MS (91.3% vs. 36.0%, *P*<0.001). On the axial section of the brain stem, the majority (82.4%) of patients with ADEM showed lesions on the ventral part; the brain stem lesions in patients with NMO were typically located in the dorsal part (91.3%); and lesions in patients with MS were found in both the ventral (44.0%) and dorsal (56.0%) parts. The lesions in patients with ADEM (100%) and NMO (91.3%) had poorly defined margins, while lesions of patients with MS (76.0%) had well defined margins. Brain stem lesions in patients with ADEM were usually bilateral and symmetrical (82.4%), while lesions in patients with NMO (87.0%) and MS (92.0%) were asymmetrical or unilateral.

**Conclusions:**

Brain stem lesions showed various morphological features among adult patients with ADEM, NMO, and MS. The different lesion locations may be helpful in distinguishing these diseases.

## Introduction

Idiopathic inflammatory demyelinating diseases (IIDDs) represent a broad spectrum of central nervous system disorders that cannot be completely differentiated on the basis of clinical course, lesion distribution on imaging, and laboratory findings [1]–[4]. This spectrum mainly includes multiple sclerosis (MS), neuromyelitis optica (NMO), and acute disseminated encephalomyelitis (ADEM). ADEM is typically known as a monophasic inflammatory demyelinating disorder with polyfocal and aggressive neurological deficits; it usually follows a viral infection or immunization [5]–[7]. NMO is an inflammatory demyelinating disease characterized by a severe acute transverse myelitis with bilateral simultaneous or sequential optic neuropathy; it results in paraplegia and blindness with or without recovery [8]–[10]. NMO is frequently reported in the Asian population, including the Chinese [9]. MS occurs in young adults with Caucasian predilection and is clinically characterized by episodes of focal disorders of the optic nerves, brain, and spinal cord; it results in characteristic complexes of symptoms and signs [11], [12].

Numerous studies have reported that magnetic resonance imaging (MRI) of patients with ADEM, NMO, and MS can show cerebrum, brain stem, cerebellum, and spinal cord lesions. Although MRI is one of the most important diagnostic criteria that help to differentiate these diseases, there is no single MRI feature that distinguishes ADEM from NMO, MS, and cerebrovascular disease [13].

Brain stem lesions on MRI were reported in 37.5% to 65.0% of patients with ADEM [5], [14]–[17], 23.0% to 44.8% of patients with NMO [9], [18], [19], and 29.0% to 58.0% of patients with MS [5], [17], [20]–[22]. However, few systemic studies have focused on brain stem lesions on MRI, especially in adult patients. Therefore, this study investigated and compared brain stem lesions on MRI in adult Chinese patients with ADEM, NMO, and MS.

## Methods

### Ethics Statement

This research was approved by the ethics committee of the Third Affiliated Hospital of Sun Yat-sen University (No. 2007-33). All participants involved in this study provided written informed consent.

### Patients

Our database comprised 282 patients with demyelinating diseases who were diagnosed and managed from 1995 to 2010 in the MS center of the Third Affiliated Hospital of Sun Yat-sen University [23]. Of 282 patients with IIDDs, 65 fulfilled the inclusion criterion, which was the presence of a brain stem lesion as detected by brain MRI. Clinical data and MRI scans were collected from these adult individuals (onset age ≥18 years), which comprised 17 patients with ADEM, 23 with NMO, and 25 with MS who were diagnosed and followed up between 2005 and 2010. ADEM was defined as a first acute fulminant demyelinating episode without any previous neurologic history. Patients who presented with acute neurologic symptoms and multiple supratentorial and/or infratentorial demyelinating lesions on MRI that were suggestive of a first and severe acute inflammatory process, as usually described in ADEM, were included in our study [15], [21], [24]. They did not meet the 2006 Wingerchuk criteria for NMO or the 2005 McDonald criteria for MS. In addition, there were no clinical relapses within 12 months of follow-up, which commenced from the date of onset. NMO was diagnosed according to the 2006 Wingerchuk criteria [25]. Patients with MS had relapse-remitting MS according to the 2005 McDonald criteria [26].

Laboratory tests were performed in all cases to exclude connective tissue diseases, infectious diseases, vascular diseases, and metabolic disorders. The cerebrospinal fluid (CSF) oligoclonal bands (OCB) detection method used in our laboratory is an isoelectric focusing technique combined with the avidin-biotin-peroxidase complex method [27]. Serum from all included patients was tested for NMO-IgG antibody by a commercial sampling kit (Euroimmun, Germany) according to the manufacturer's instructions. All participants involved in this study provided written informed consent.

### MRI scanning

Brain and spinal cord MRI scans were performed in all patients using a GE 1.5T MR scanner (General Electric, Milwaukee, WI, USA). The slice thickness of the axial scans was between 3 and 5 mm. Conventional MRI protocols were used in all patients: T1 with and without gadolinium enhancement (400/15.5 ms, TR/TE) and T2 (2500–3500/100 ms, TR/TE) in spinal cord MRI; and T1 with and without gadolinium enhancement (2128–2300/11.6–12.4 ms, TR/TE), T2 (4600–4640/97.8–102 ms, TR/TE), and fluid-attenuated inversion recovery (FLAIR) (8800/120 ms, TR/TE) in brain MRI. All MRI scans were performed prior to corticosteroid treatment. No patients were receiving interferon-beta or immunosuppressants at the time of MRI scanning. The total brain stem lesion number, location, and pattern of lesion distribution were recorded along with the size of the largest lesions. All image archives were reviewed with a DICOM Viewer (OsiriX v3.2.1, http://www.osirix-viewer.com) on a Macintosh computer. All MRI scans were analyzed by one experienced neuroradiologist (Z.K.) and one neurologist (W.Q.) who were blinded to the diagnostic categorization and the patients' clinical features.

### Statistical analysis

Statistical analysis was performed by SPSS version 13.0. *P* values of <0.05 were considered statistically significant. Quantitative data were processed using the Mann–Whitney U test, one-way analysis of variance (ANOVA), rank of one-way ANOVA, or pairwise comparison among groups with the least significant difference (LSD) test (level of test *α* = 0.05). All quantitative data in this study are presented as mean ± standard deviation (SD) or median ± range. Qualitative data were analyzed with the chi-square test or Fisher's exact test.

## Results

### Clinical and laboratory features

The clinical and laboratory features of the patients with ADEM, NMO, and MS are summarized in Table 1. There were no statistical differences in female/male ratios or ages at onset among these three groups (*P*>0.05). Patients with ADEM had a shorter disease duration than did patients with NMO (4.0 vs 36.0 months, *P*<0.001) and MS (4.0 vs 24.0 months, *P*<0.001). Patients with ADEM displayed a greater Expanded Disability Status Scale (EDSS) score at their last visit than did patients with NMO (5.0 vs 3.5, *P* = 0.015). The EDSS score of patients with ADEM was also greater than that of patients with MS (5.0 vs 3.0, *P*<0.001).

**Table 1:** Demographic and clinical characteristics of patients with ADEM, NMO, and MS.

	ADEM (n = 17)	NMO (n = 23)	MS (n = 25)	*P*	*P*1	*P*2	*P*3
Gender, F∶M	7∶10	18∶5	15∶10	0.057	–	–	–
Age at onset, years	30.2±13.3(18–58)	38.4±12.5(18–60)	37.0±12.4(18–60)	0.115	–	–	–
Disease duration, months	4.0(1.0–10.0)	36.0(15.0–84.0)	24.0(12.0–156.0)	<0.001	<0.001	<0.001	0.844
Follow-up, months	18.0(13.0–36.0)	27.0(15.0–41.0)	24.0(12.0–45.0)	0.020	0.010	0.016	0.803
Relapse number	–	5.0 (2.0–12.0)	3.0(2.0–12.0)	–	–	–	0.008
EDSS at last visit	5.0(2.5–10.0)	3.5(1.5–6.5)	3.0 (1.0–5.5)	0.002	0.015	<0.001	0.204
Clinical features, n(%)							
Fever	7(41.2)	0	0	<0.001	0.003	0.002	–
Headache	4(23.5)	4(17.4)	1(4.0)	0.13	–	–	–
Meningismus	5(29.4)	0	0	0.001	0.022	0.016	–
Encephalopathy	9(52.9)	1(4.3)	1(4.0)	<0.001	0.002	0.001	1
Seizure	3(17.6)	0	0	0.014	0.137	0.117	–
Pyramidal	16(94.1)	17(73.9)	20(80.0)	0.207	–	–	–
Sensory	9(52.9)	21(91.3)	19(76.0)	0.021	0.016	0.12	0.301
Extrapyramidal	1(5.9)	1(4.3)	3(12.0)	0.584	–	–	–
Visual	1(5.9)	23(100.0)	5(20.0)	<0.001	<0.001	0.404	<0.001
Brainstem	15(88.2)	18(78.3)	24(96.0)	0.158	–	–	–
Myelitis	11(64.7)	21(91.3)	12(48.0)	0.006	0.093	0.286	0.001
CSF OCB (+),n (%)	2(11.8)	6(26.1)	7(28.0)	0.431	–	–	–
NMO-IgG(+),n (%)	0	14(60.9)	3(12.0)	<0.001	<0.001	0.383	<0.001

Abbreviations: ADEM = acute disseminated encephalomyelitis; NMO = neuromyelitis optica; MS = multiple sclerosis; *P* = comparative three groups; *P*1 = ADEM vs NMO; *P*2 = ADEM vs MS; *P*3 = NMO vs MS; F = female; M = male; EDSS = Expanded Disability Status Scale; CSF = cerebrospinal fluid; OCB = oligoclonal bands.

Fever, meningismus, and encephalopathy were more commonly seen in patients with ADEM than in patients with NMO and MS. Meanwhile, clinical features of optic neuritis and myelitis were more frequent in patients with ADEM than in patients with NMO and MS.

NMO-IgG seropositivity was significantly higher in patients with NMO than in patients with ADEM and MS (*P*<0.001). There was no statistical difference in OCB positivity of CSF among these three groups (*P* = 0.431). OCB positivity in MS patients in this cohort was 28.0%, which is consistent with other reports on Asian populations, varying from 3.3% to 33.3% [28]–[31].

### Brain stem lesions

**Figure 1:**
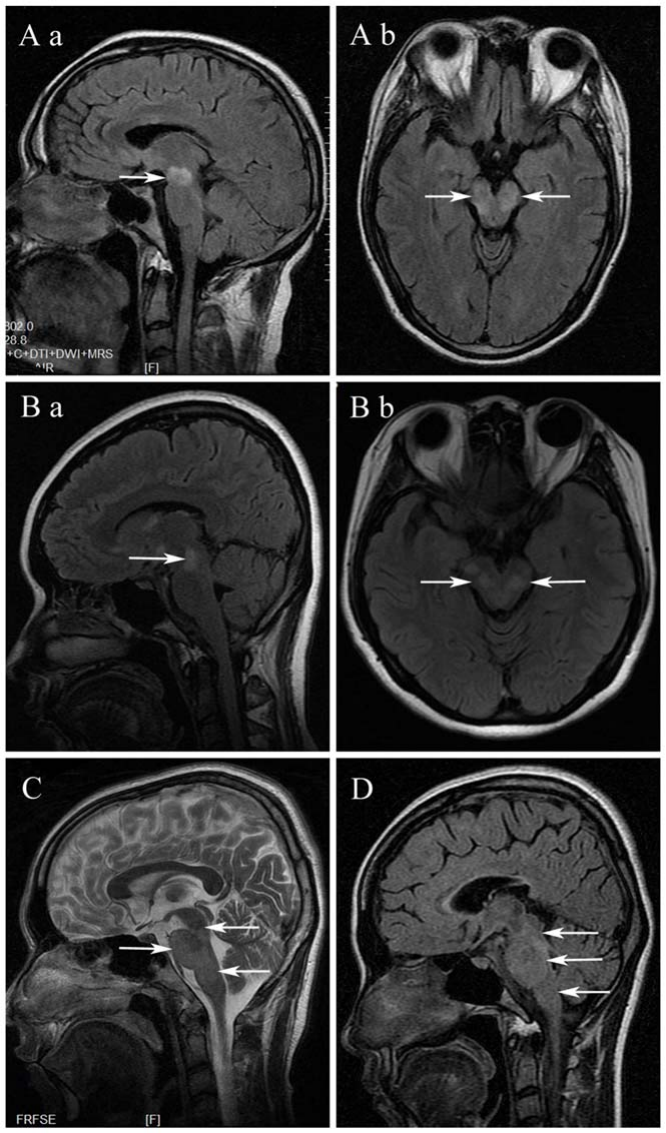
Brain stem lesions (arrows) on MRI of acute disseminated encephalomyelitis (ADEM). (A, B) Fluid-attenuated inversion recovery (FLAIR) image showing midbrain lesions with poorly defined margins located in the ventral part (symmetrical or bilateral). (C, D) Sagittal image highlighting multiple brain stem lesions (midbrain, pons, medulla) with poorly defined margins.

As shown in Figures 1–3 and Table 2, patients with ADEM had more brain stem lesions than did patients with NMO (3 [1–6] vs 1 [1–5], *P*<0.001) and MS (3 [1–6] vs 2 [1–3], *P*<0.001). However, there was no statistical difference between patients with NMO and those with MS (*P* = 0.314). Lesion size in patients with MS was significantly smaller than that in patients with ADEM (8.0 [5.0–17.0] vs 10.0 [8.0–26.0] mm, *P* = 0.009) and NMO (8.0 [5.0–17.0] vs 12.0 [3.0–17.0] mm, *P* = 0.001).

**Figure 2 pone-0022766-g002:**
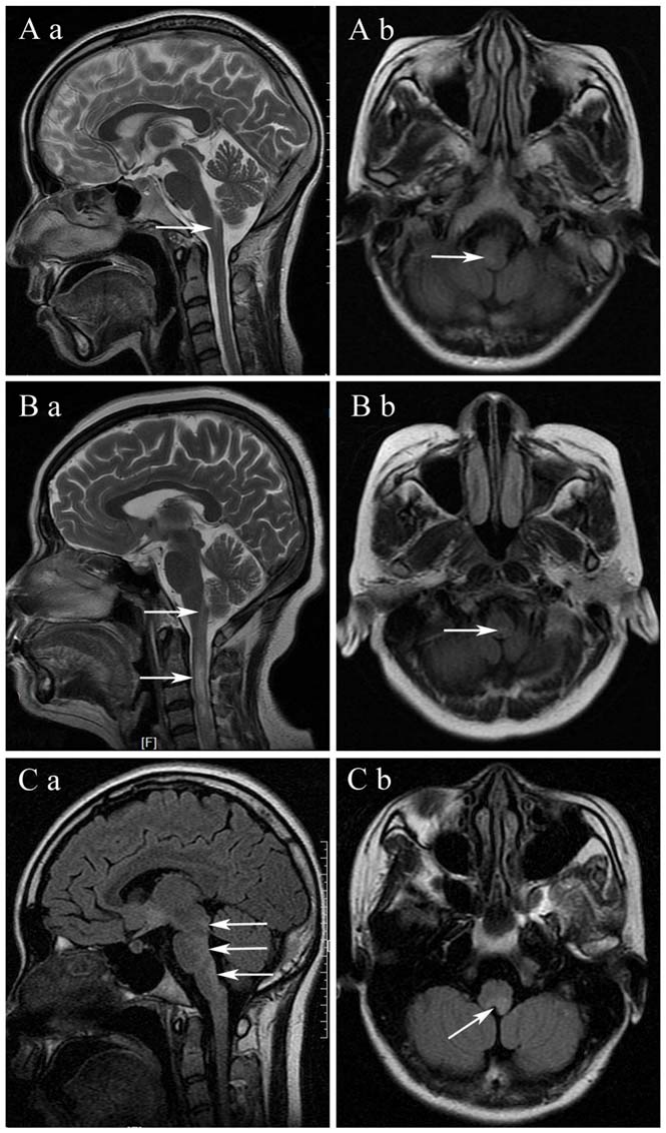
Brain stem lesions (arrows) on MRI of neuromyelitis optica (NMO). (A) MRI showing medulla lesion located in the dorsal part with poorly defined shape. (B) MRI showing linear medullospinal lesions. (C) MRI showing distribution of NMO-characteristic brain lesions (dorsal brain stem) corresponding to sites of high NMO-IgG expression.

**Figure 3:**
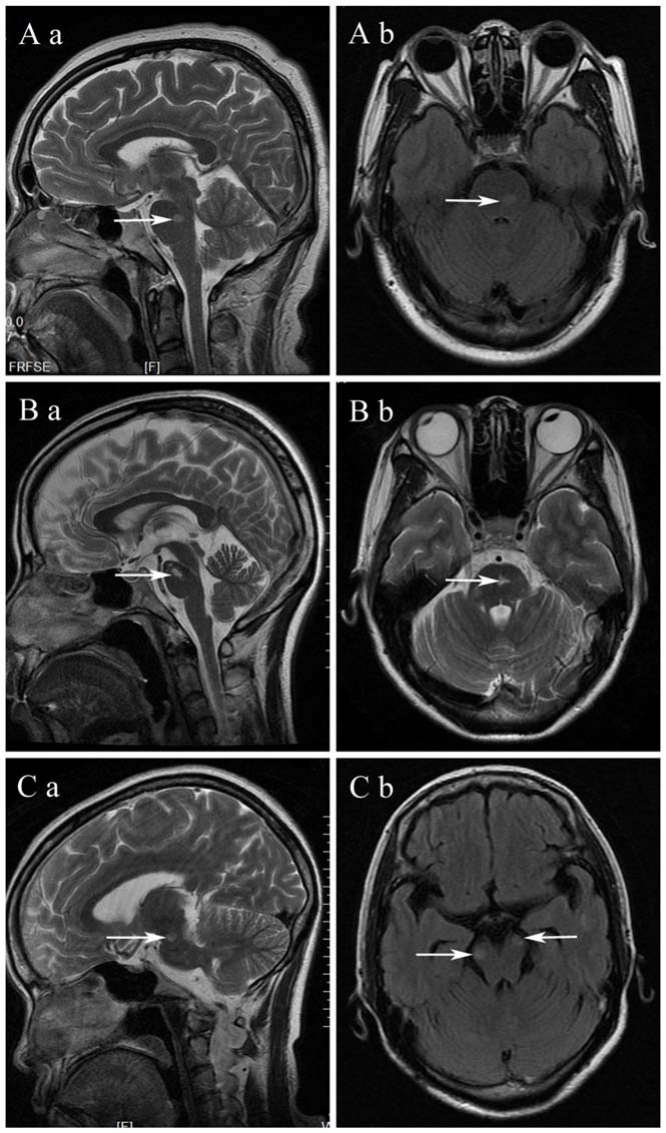
Brain stem lesions (arrows) on MRI of multiple sclerosis (MS). (A, B) MRI showing solitary pons lesion with sharply defined borders. (C) MRI showing midbrain lesions.

**Table 2:** Comparative brain stem lesions on MRI in patients with ADEM, NMO, and MS.

	ADEM (n = 17)	NMO (n = 23)	MS (n = 25)	*P*	*P*1	*P*2	*P*3
Number of lesions	3(1–6)	1(1–5)	2(1–3)	<0.001	<0.001	<0.001	0.314
Sagittal section							
Diameter, median, mm	10.0(8.0–26.0)	12.0(3.0–17.0)	8.0(5.0–17.0)	0.030	0.726	0.009	0.001
Midbrain, n (%)	16 (94.1)	4 (17.4)	10(40.0)	<0.001	<0.001	<0.001	0.085
Pons, n (%)	12 (70.6)	8 (34.8)	21 (84.0)	0.001	0.025	0.511	<0.001
Medulla, n (%)	6 (35.3)	21(91.3)	9 (36.0)	<0.001	<0.001	0.963	<0.001
Axial section							
Ventral, n (%)	14 (82.4)	2 (8.7)	11(44.0)	<0.001	<0.001	0.013	0.006
Dorsal, n (%)	3 (17.6)	21 (91.3)	23 (56.0)	<0.001	<0.001	0.013	0.006
Well defined margins, n (%)	0	2 (8.7)	19 (76.0)	<0.001	0.499	<0.001	<0.001
Bilateral or symmetrical, n (%)	14 (82.4)	3 (13.0)	2 (8.0)	<0.001	<0.001	<0.001	0.572

Abbreviations: ADEM = acute disseminated encephalomyelitis; NMO = neuromyelitis optica; MS = multiple sclerosis; *P* = comparative three groups; *P*1 = ADEM vs NMO; *P*2 = ADEM vs MS; *P*3 = NMO vs MS.

Patients with ADEM had a significantly higher frequency of midbrain lesions than did patients with NMO (94.1% vs 17.4%, *P*<0.001) and MS (94.1% vs 40.0%, *P*<0.001). However, there was no statistical difference in midbrain lesions between patients with NMO and those with MS (17.4% vs 40.0%, *P* = 0.085). Patients with NMO had a significantly lower frequency of pons lesions than did patients with MS (34.8% vs 84.0%, *P*<0.001) and ADEM (34.8% vs 70.6%, *P* = 0.025). There was no statistical difference in pons lesions between patients with ADEM and MS (70.6% vs 84.0%, *P* = 0.511). Patients with NMO had a significantly higher frequency of medulla oblongata lesions than did patients with ADEM (91.3% vs 35.3%, *P*<0.001) and MS (91.3% vs 36.0%, *P*<0.001). However, there was no statistical difference in medulla oblongata lesions between patients with ADEM and those with MS (35.3% vs 36.0%, *P* = 0.963).

On the axial section of brain MRI, the majority (82.4%, 14/17) of patients with ADEM showed lesions on the ventral part, while the brain stem lesions in patients with NMO were typically located in the dorsal part (91.3%, 21/23). Lesions in patients with MS were found in both the ventral (44.0%) and dorsal (56.0%) parts. Lesions in patients with ADEM (100%) and NMO (91.3%) had poorly defined margins, contrary to the lesions in patients with MS (76.0%), which showed well-defined margins.

Furthermore, brain stem lesions in patients with ADEM were usually symmetrical and bilateral (82.4%, 14/17, *P*<0.001), while lesions in patients with NMO (87.0%, 20/23) and MS (92.0%, 23/25) were usually asymmetrical or unilateral.

### Brain and spinal cord lesions

Cerebrum, cerebellum, and spinal cord lesions are shown in Table 3. Cortical gray matter and deep gray matter lesions (basal ganglion) were most commonly seen in patients with ADEM, while lesions in the subcortical white matter, periventricular area, and corpus callosum were common in patients with MS. T1 black hole lesions were also more frequent in patients with MS than in those with ADEM or NMO. All enrolled patients had repeated brain MRI scans; there were no new lesions in patients with ADEM, while new brain lesions were found in patients with NMO and MS. All patients with NMO, 52.9% of patients with ADEM, and 44.0% of patients with MS showed spinal cord lesions.

**Table 3:** Comparative brain and spinal cord lesions on MRI in patients with ADEM, NMO, and MS.

	ADEM (n = 17)	NMO (n = 23)	MS (n = 25)	*P*	*P*1	*P*2	*P*3
Cerebrum							
Number of lesions >10, n(%)	5(29.4)	0	15(60.0)	<0.001	0.022	0.089	<0.001
Diameter in axial section, mm	23.2±12.5	12.4±7.0	16.0±5.8	0.001	<0.001	0.072	0.024
Cortical gray matter, n(%)	10(58.8)	2(8.7)	2(8.0)	<0.001	0.001	0.001	1.000
Deep gray matter, n(%)	15(88.2)	8(34.8)	5(20.0)	<0.001	0.001	<0.001	0.250
Subcortical white matter, n(%)	9(52.9)	9(39.1)	22(88.0)	0.002	0.385	0.029	<0.001
Deep white matter, n(%)	16(94.1)	17(73.9)	24(96.0)	0.048	0.214	1.000	0.079
Periventricular white matter, n(%)	7(41.2)	5(21.7)	24(96.0)	<0.001	0.185	<0.001	<0.001
Corpus callosum, n(%)	3(17.6)	2(8.7)	20(80.0)	<0.001	0.717	<0.001	<0.001
Cerebellum,n(%)	7(41.2)	2(8.7)	16(64.0)	<0.001	0.040	0.145	<0.001
T1 black hole, n(%)	2(11.8)	0	14(56.0)	<0.001	0.340	0.004	<0.001
Gadolinium enhancement, n (%)	12(70.6)	3(13.0)	10(40.0)	0.001	<0.001	0.051	0.036
Well defined margins, n (%)	2(11.8)	1(4.3)	20(80.0)	<0.001	0.785	<0.001	<0.001
Repeated brain MRI, n (%)	17(100.0)	23(100.0)	25(100.0)	–	–	–	–
New lesions, n (%)	0	8(34.8)	24(96.0)	<0.001	0.020	<0.001	<0.001
Spinal cord, n (%)	9(52.9)	23(100.0)	11(44.0)	<0.001	0.001	0.569	<0.001
Lesion length, Segments	7.6±4.9	8.0±5.1	4.8±3.5	0.183	–	–	–
Gadolinium enhancement, n (%)	4/9(44.4)	13/23(56.5)	7/11(63.6)	0.687	–	–	–
Cervical segment,n (%)	4/9(44.4)	8/23(34.8)	4/11(36.4)	–	–	–	–
Thoracic segment, n (%)	3/9(33.3)	2/23(8.7)	4/11(36.4)	–	–	–	–
Combination, n(%)	2/9(22.2)	13/23(56.5)	3/11(27.3)	–	–	–	–

Abbreviations: ADEM = acute disseminated encephalomyelitis; NMO = neuromyelitis optica; MS = multiple sclerosis; *P* = comparative three groups; *P*1 = ADEM vs NMO; *P*2 = ADEM vs MS; *P*3 = NMO vs MS.

## Discussion

Brain stem lesions on MRI have been reported in patients with ADEM, NMO, and MS [15], [20]. However, few studies have focused on comparison of the brain stem among these three diseases. To our knowledge, this is the first report comparing brain stem lesions on MRI in adult patients with ADEM, NMO, and MS. In the present study, we found the following distinguishing features of brain stem lesions on MRI in adult patients with ADEM, NMO, and MS: midbrain lesions in the ventral part with poorly defined margins for ADEM, medulla lesions in the dorsal part with poorly defined shape for NMO, and pons lesions with well-defined shape for MS.

In adult patients with ADEM, midbrain lesions (94.1%) were more common than pons (70.6%) and medulla oblongata (35.3%) lesions. This implies that the location of brain stem lesions differs between adult and pediatric patients with ADEM; brain stem lesions have been shown to be more frequently involved in the pons in pediatric patients with ADEM [20]. Furthermore, we found that lesions in patients with ADEM were usually located in the ventral part with poorly defined margins. It can be speculated that ADEM lesions may first involve the cerebrum, then disseminate to the midbrain, and finally involve the spinal cord.

Lesions in the brain stem were relatively characteristic of NMO, showing involvement of the brain stem in continuity with cervical cord abnormalities [8], [32]. Our results were consistent with those of previous reports showing that the medulla is the most common lesion location in NMO, and lesions are usually located in the dorsal part with poorly defined shapes. The distribution of NMO-characteristic brain stem lesions corresponds to sites of high aquaporin-4 protein expression [32].

Our study has shown that MS lesions often occur in the pons within the brain stem, similar to the reports by Brainin and Triulzi [33], [34]. Furthermore, we found that solitary brain stem lesions in the dorsal or ventral part with sharply defined borders are commonly seen in patients with MS.

Several MRI criteria have been proposed for differentiating ADEM from MS in children [35]–[38], of which the Callen MS-ADEM criterion has the highest sensitivity and specificity [39]. In the present study, however, two adult patients with ADEM (11.8%) had T1 black hole lesions, and seven patients (41.2%) had periventricular white matter lesions that were parallel to the lateral ventricles. Therefore, the Callen MS-ADEM criterion may not be suitable for adults with ADEM. To our knowledge, only two studies have focused on ADEM in adults [15], [21]. Our findings (disseminated, symmetrical, or bilateral and commonly located in the midbrain and ventral part with poorly defined margins) will be helpful for differential diagnosis in adults with ADEM.

We acknowledge that our study has some limitations: (1) the follow-up duration of the enrolled patients was relatively short; (2) because of the exclusion of children and patients without brain stem lesions, our conclusion should not be applied to these patients; and (3) as a retrospective study, bias is inevitable.

In conclusion, brain stem lesions on MRI showed various morphological features among adult patients with ADEM, NMO, and MS. The different lesion locations in the brain stem may be helpful in distinguishing these diseases.
